# Reproducibility and concordance of functional autonomy tests in older adult women: a comparative study of face-to-face and virtual assessments

**DOI:** 10.3389/fpubh.2024.1445039

**Published:** 2025-01-24

**Authors:** Talles M. Siqueira, Rafael M. Pitta, Alexandre F. Machado, Fabiana R. Scartoni, Roberta L. Rica, Francisco Luciano Pontes Junior, Valentina Bullo, Stefano Gobbo, Marco Bergamin, Danilo S. Bocalini

**Affiliations:** ^1^Experimental Physiology and Biochemistry Laboratory, Physical Education and Sport Center of Federal University of Espirito Santo, Vitoria, Brazil; ^2^Postgrad Program Health Science, Instituto Israelita de Ensino & Pesquisao, São Paulo, Brazil; ^3^Sport Exercise Sciences Laboratory - LaCEE, Catholic University of Petrópolis, Petrópolis, Brazil; ^4^Departament of Physical Education, Estácio de Sá University, Vitoria, Brazil; ^5^Exercise Physiology and Aging Laboratory-LaFEE, School of Arts, Sciences and Humanities, University of São Paulo, São Paulo, Brazil; ^6^Department of Medicine, University of Padova, Padova, Italy

**Keywords:** aged, older people, longevity, daily living activity, older adults

## Abstract

**Introduction:**

The literature does not explore functional assessments carried out remotely and in older women in virtual environments.

**Objective:**

This study analyzed the reproducibility and agreement in applying functional autonomy tests face to face (FF) and virtually (V).

**Methods:**

A single evaluator carried out two evaluations. The following tests were performed: walking 10 m, rising from the sitting position (RSP), rising from the ventral decubitus position (RVDP), and sitting and rising from a chair and walking around the house (SRCW).

**Results:**

No significant changes were identified between V and FF (*p* > 0.05 for all). No significant changes were identified between tests considering FF and V conditions (*p* > 0.05 for all). The highest value for the intraclass correlation coefficient was <0.0001 for the SRCW (CL, *r* = 0.98 CI95%: 0.969–0.990 and ICC, *r* = 0.99 CI95%: 0.984–0.995), and the lowest was <0.0001 for the RSP (CL, *r* = 0.91 CI95%: 0.853–0.954 and ICC, *r* = 0.95 CI95%: 0.921–0.976). Regarding agreement between tests, a variation was found between the lowest value of 0.07 ± 0.74 BIAS for the RVDP and the highest value of 0.32 ± 1.89 BIAS for the SRCW.

**Conclusion:**

The tests used in the present study showed good reproducibility and agreement in older people when carried out face to face and virtually.

## Introduction

The demographic profile of the older adult population has undergone significant changes in recent years related to increased life expectancy, with an increasing number of people aged 60 or over. In 2019, older people numbered more than 1 billion worldwide, with projections for 2030 being 1.4 billion and for 2050, 2.1 billion. There are more older people than children under five, demonstrating an inversion in the age pyramid, which also presents a decrease in birth rates, especially in developing countries (1).

Inevitably, the aging process is associated with physiological changes and musculoskeletal changes that cause progressive declines in the function of biological systems (2, 3) as well as the presence of chronic or locomotor conditions, which, in addition to the risk to life, represent a potential threat to the independence and autonomy of movement of older adults. Therefore, to age healthily, it is necessary to focus on maintaining and/or improving multiple health variables such as range of movement, muscular strength, balance, cardiorespiratory endurance, joint mobility, and agility, among others (4–6).

Both physical activity and physical exercise are effective non-pharmacological methods for reducing physical disability, in addition to helping to reduce the risk of various chronic diseases (7–11) recognized as disabling in advanced clinical stages (9, 12–14).

Given this context, the wellbeing, health maintenance, and quality of life of the older adult population are of fundamental importance in global public health. In this regard, considering the importance of functional autonomy in aging well, tests have been developed and validated to analyze functional autonomy. These tests help establish an accurate diagnosis and help support recommendations (whether physical, mental, or social) that contribute to the overall ability of an older person to function effectively in daily life.

With the advent of the COVID-19 pandemic, which was recognized by the World Health Organization (WHO) on 11 March 2020, several guidelines were established for developing occupational activities in the context of the home environment (15, 16). Social restriction was the most widely implemented measure by authorities, resulting in significant changes in citizens’ lifestyles and mental health (17). These social restrictions were associated with reductions in physical activity levels and increases in sedentary behavior (18–21). In parallel with social restrictions, there was a considerable increase in physical activity programs delivered remotely using technology, a strategy that has continued to be employed for providing care to individuals with numerous conditions. The unprecedented aspects of the present study are framed within two contexts: first, the continuity of physical exercise practice regardless of social isolation, and second, the need for diagnostic evaluation to support such practices. These aspects highlight the relevance of the present study. To ensure a healthy exercise prescription, continuous evaluation and monitoring of key parameters are essential.

However, the actual effectiveness, reliability, and reproducibility of functional assessments carried out remotely in a virtual environment remain unclear in the literature. Given that physical exercise is an essential tool for reducing the harmful effects of confinement and increasing immunity in older adults, it became necessary to adapt diagnostic assessment instruments for use in the virtual environment. However, the literature presents gaps related to the efficiency of these assessments. To verify the efficiency of applying tests virtually, the objective of the present study was to analyze the reproducibility and agreement in applying functional autonomy tests face to face and virtually for older people. This study anticipates reproducibility and agreement in virtual tests of functional autonomy in older women.

## Materials and methods

After approval by the Ethics and Research Committee of the Federal University of Espirito Santo (n° 5.029.735/2022), older women were invited to participate in the study. The invitation was circulated in community centers, squares, parks, and gyms through posters and leaflets, as well as through social networks.

### Participants

The inclusion criteria were being 60 or older, physically active, and independent in daily activities. As a non-inclusion criterion, any veto was adopted from clinical examinations or pre-participatory assessments carried out by health professionals. The exclusion was applied to acute or chronic conditions that could compromise or become an impediment to carrying out the tests, such as recent hospitalization, symptomatic cardiorespiratory disease, uncontrolled hypertension or metabolic syndrome, severe kidney or liver disease, cognitive impairment or progressive and debilitating conditions, severe obesity with inability to perform physical activity and recent bone fractures. Older people who did not perform all tests in virtual and face-to-face conditions were excluded from the sample. Initially, 60 older women came forward to participate in the tests. However, after applying the non-inclusion and exclusion criteria, 44 older women were considered eligible to participate.

### Test protocols

**Figure 1:**
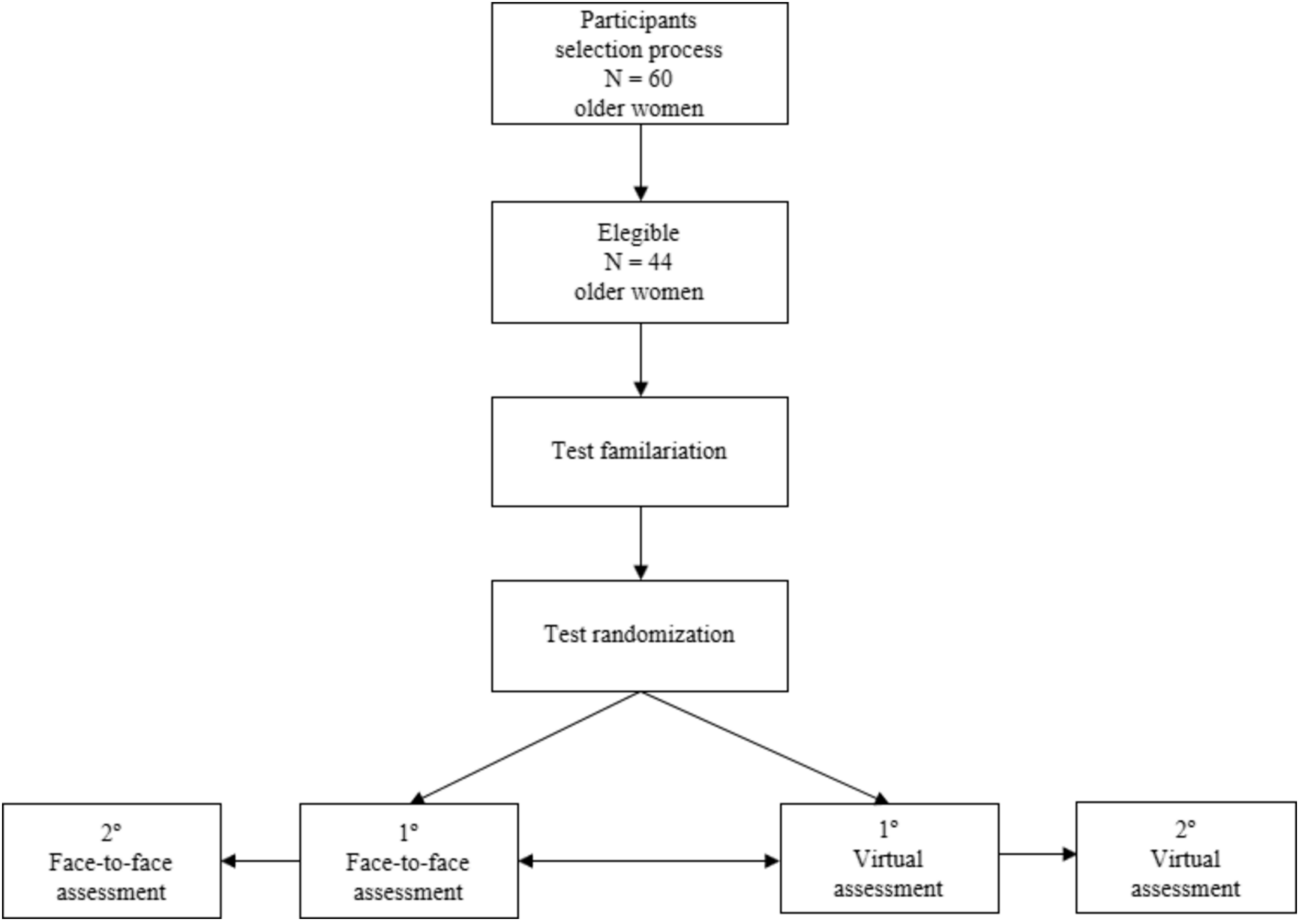
Flowchart of experimental design.

The applications of functional autonomy tests, virtually and face to face, were randomly distributed using a randomization program with 48-h intervals between them. After familarization, with both tests, two assessments were conducted to analyze the test and retest. Four evaluations were similarly carried out by a single evaluator with experience in the procedures applied. The face-to-face and virtual assessments were carried out in the same environment between both conditions. A conventional microcomputer with Internet access and high resolution was used to carry out the virtual assessment. Figure 1 presents a summary of the study design.

### Parameters evaluated

#### Anthropometric data

To measure body mass and height, the participant was required to be barefoot and wearing physical activity clothing (light clothes, shorts, and a shirt). They stood on the central part of the platform of the Filizola® mechanical scale (Brazil) with INMETRO seal, having an accuracy of 100 g. Weight was measured in kilograms.

The standard previously mentioned was used to measure height with a Cardiomed^®^ WCS model stadiometer, with an accuracy of 1 mm. The participant was instructed to stand upright, with their arms extended along their body and feet together, holding their breath in inspiratory apnea. The head was oriented according to the Frankfurt plane, ensuring the measurement was taken in centimeters. Body mass index (BMI, kg/m ^2^) was calculated according to the formula: weight/height^2^.

### Functional autonomy tests

The Latin American Development Group for Maturity (GDLAM) protocol aims to evaluate the functional autonomy of older adults and can be used by professionals in specific areas of health for diagnosis and control of autonomy. It is widely used by researchers from all parts of the world (22).

The following tests assessed functional autonomy: walking 10 m (W10m), which assesses speed, where the individual needs to walk as quickly as possible within 10 m. The objective is to assess gait speed. The participant standing next to the start demarcation, at the command of “Now,” walks as quickly as possible within 10 m, and only stops walking when they pass the indicated demarcation. The test area consists of a marking of 10 m (test space) and one of 15 m (5 m of space for deceleration). The test was carried out on flat, ventilated, and illuminated ground to ensure the participant’s safety (23).

Rising from a sitting position (RSP). The objective is to assess the functional capacity of the lower limbs. The test begins with the participant sitting in a chair, with arms crossed in front of the chest (so that there is no help from the hands). At the command of “Now,” the participant needs to sit down and stand up correctly five times as fast as they can. The test ends with the participant seated in a chair with a back but no arms, with the seat height measuring 46–48 cm from the ground (24). Rising from *de ventral decubitus* position (RVDP). The objective is to assess the participant’s ability to get up from the floor as quickly as possible. The test starts with the participant lying face down in the prone position, arms extended alongside the body and supported by the mat, with palms facing upward. Upon hearing the command “Now,” the participant rises from the floor as quickly as possible and stands up. The test also involves sitting and standing from a chair and walking around the house (SRCW) (25), aiming to evaluate agility, dynamic balance, and balance recovery. The test begins with the participant sitting with their feet suspended. At the command of “Now,” they get up from the chair, move to one of the cones, circle it, return toward the chair and sit down (always taking their feet off the floor when sitting), get up, and repeat the process for the other side, and do this once again for both sides, that is, the process repeats itself twice. The test ends with the participant seated (26). All tests are measured in seconds, and the results are used to calculate the general functional autonomy index (GI). All tests were performed in the order described above, on a single day, using a 3-min interval between them to allow good recovery between tests (22).

### Statistical analysis

The Shapiro–Wilk test was used to check data normality. Student’s *t*-test was used to verify the differences in the means of the functional aptitude tests between the first and second assessments. The typical absolute and relative measurement errors of all parameters were calculated following the model proposed by Perini et al. (27). Agreement between measurements was analyzed using linear correlation, with weak (< 0.4), moderate (> 0.4 and < 0.5), and strong (≥ 0.5) correlations as interpretations. Reproducibility was determined by the two-way intraclass correlation coefficient being interpreted as little correlation (<0.25), low correlation (> 0.26 to <0.49), moderate (> 0.50 to <0.69), high (> 0.7 to <0.89), and very high (> 0.9 to <1.0). The reliability between the measures was analyzed using the Bland and Altman graphical arrangement. The effect size was calculated using Hedges’ g, with values between 0.2 and 0.5 being interpreted as small, 0.5 and 0.8 as moderate, and values above 0.8 as significant. All statistical analyses were performed using the GraphPad Prism software (version 4.0, San Diego, CA, United States) with a significance level of *p* < 0.05, with data presented as mean ± standard deviation, coefficient of variation, and differences between means and 95% confidence interval.

## Results

The older women were 67 ± 5 years old, 1.57 ± 0.5 m in height, and had a body mass index of 28 ± 3 kg/m^2^. The results of the test and retest assessments of the older women’s functional autonomy parameters are described in Table 1.

**Table 1:** Test and retest analyses of the functional assessment parameters of older people in a face-to-face and virtual environment (*n* = 44).

Tests	Condition	1st Assessment	2nd Assessment	MD	95% CI	TE	*t*	*P*	Absolute ETM	Relative ETM
W10m	Face to face	7.06 ± 2.37 (33.62%)	7.02 ± 2.25 (32.13%)	0.04	−0.137–0.046	0.30	1,000	0.322	0.04	0.56%
	Virtual	7.20 ± 2.13 (29.57%)	7.11 ± 2.03 (28.64%)	0.09	−0.249–0.067	0.52	1,159	0.252	0.04	0.57%
RSP	Face to face	8.22 ± 2.67 (32.52%)	8.25 ± 2.60 (31.60%)	0.02	−0.179–0.224	0.67	0.226	0.821	0.02	0.27%
	Virtual	8.36 ± 2.66 (31.91%)	8.36 ± 2.62 (31.38%)	0.00	−0.146–0.146	0.48	0.000	0.999	0.01	0.15%
RVDP	Face to face	4.88 ± 2.99 (61.35%)	5.04 ± 3.04 (60.29%)	0.16	−0.047–0.365	0.68	1,552	0.128	0.06	1.17%
	Virtual	4.97 ± 2.81 (56.58%)	5.02 ± 2.60 (51.79%)	0.04	−0.150–0.241	0.65	0.467	0.642	0.03	0.58%
SRCW	Face to face	30.98 ± 10.57 (34.11%)	31.00 ± 10.14 (32.70%)	0.02	−0.150–0.241	1.05	0.144	0.886	0.03	0.10%
	Virtual	30.89 ± 10.20 (33.03%)	31.73 ± 9.72 (30.66%)	0.84	−0.021–1.703	2.86	1,967	0.057	0.14	0.44%

Values expressed as mean ± standard deviation and coefficient of variation (CV), mean difference (MD), confidence interval (CI), effect size (TE), *t*-test (t), typical measurement error (ETM) of the walking 10 m (W10m), rising from a sitting position (RSP), rising from de ventral decubitus position (RVDP), sitting and rising from a chair and walking around the house (SRCW) tests.

No significant changes were identified between the functional autonomy tests considering the first and second assessments in virtual and face-to-face conditions. The values of typical absolute measurement errors varied between the lowest value of 0.01 in the virtual condition and 0.02 in the face-to-face condition for the RSP test. The highest values of typical absolute measurement errors were 0.14 for the SRCW test in the virtual condition and 0.06 for the RVDP test in the face-to-face condition.

The values of typical relative measurement errors varied between the lowest value of 0.15% in the virtual condition for the RSP test and 0.10% in the face-to-face condition for the SRCW test. The highest values of typical absolute measurement errors were 0.58% in the virtual condition and 1.17 for the RVDP test. Table 2 presents the comparative values between the face-to-face and virtual conditions.

**Table 2:** Analysis of functional assessment parameters of older people in a face-to-face and virtual environment (*n* = 44).

Tests	Face to face	Virtual	MD	95% CI	TE	*t*	*P*	Absolute ETM	Relative ETM
W10m	6.97 ± 2.30 (33.02%)	7.13 ± 2.09 (29.35%)	0.15	−0.103–0.412	0.85	1,209	0.233	0.06	0.79%
RSP	8.23 ± 2.72 (33.09%)	8.37 ± 2.68 (32.02%)	0.14	−0.181–0.475	1.10	0.858	0.395	0.06	0.67%
RVDP	4.91 ± 3.05 (62.16%)	4.99 ± 2.72 (54.53%)	0.07	−0.148–0.307	0.75	0.704	0.484	0.04	0.84%
SRCW	30.95 ± 10.28 (33.22%)	31.28 ± 9.84 (31.48%)	0.32	−0.249–0.903	1.91	1,145	0.258	0.08	0.27%

Values expressed as mean ± standard deviation and coefficient of variation (CV), mean difference (MD), confidence interval (CI), effect size (TE), *t*-test (t), typical measurement error (ETM) of the walking 10 m (W10m), rising from a sitting position (RSP), rising from de ventral decubitus position (RVDP), and sitting and rising from a chair and walking around the house (SRCW) tests.

No significant changes were identified between the functional autonomy tests considering the face-to-face and virtual conditions. The values of typical absolute and relative measurement errors ranged from the lowest value of 0.04 for the RVDP tests and 0.27% for the SRCW test. The most significant typical absolute and relative measurement errors ranged between 0.08 for the SRCW and 0.84% for the RVDP tests. The analysis of linear correlations and intraclass correlation coefficients is presented in Table 3.

**Table 3:** Linear correlations and intraclass correlation coefficient of functional aptitude tests for older people (*n*=44).

Parameters	LC			ICC		
	*r*	95% CI	*P*	*r*	95% CI	*P*
W10m	0.93	0.874–0.961	< 0.0001	0.96	0.929–0.979	< 0.0001
RSP	0.91	0.853–0.954	< 0.0001	0.95	0.921–0.976	< 0.0001
RVDP	0.97	0.950–0.985	< 0.0001	0.98	0.969–0.991	< 0.0001
SRCW	0.98	0.969–0.990	< 0.0001	0.99	0.984–0.995	< 0.0001

Expressed values of the confidence interval (CI) of the linear correlations (LC) and the intraclass correlation coefficient (ICC) of the walking 10 m (W10m), rising from a sitting position (RSP), rising from de ventral decubitus position (RVDP), and sitting and rising from a chair and walking around the house (SRCW) tests between the face-to-face and virtual conditions.

**Figure 2:**
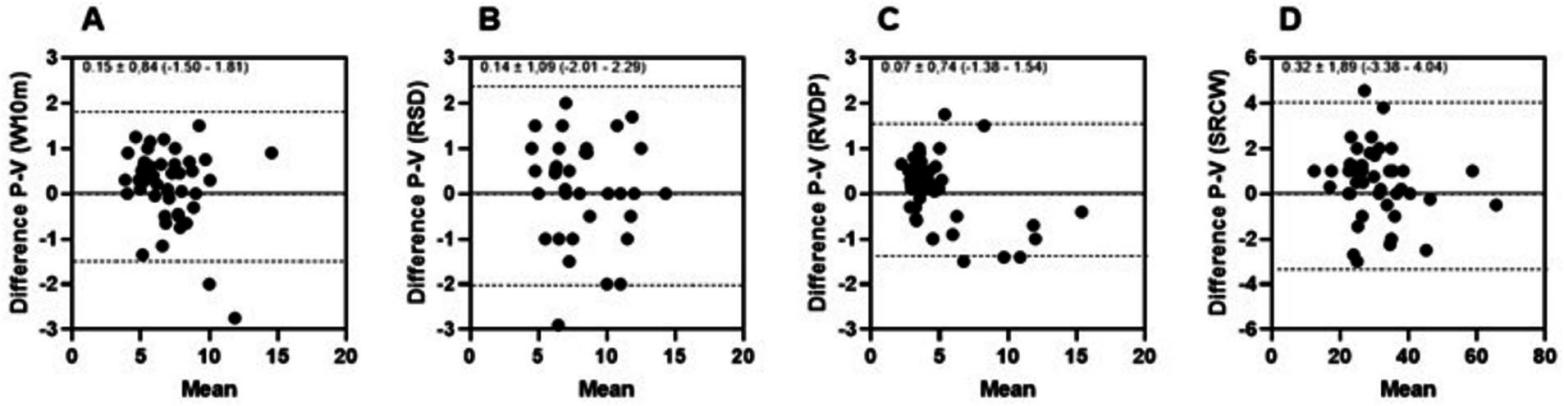
Reproducibility between measures of functional autonomy tests by Bland–Altman plot of face-to-face (P) and virtual (V) assessments of the walking 10 m (W10m) **(A)**, rising from a sitting position (RSP) **(B)**, rising from the ventral decubitus position (RVDP) **(C)**, and sitting and rising from a chair and walking around the house (SRCW) **(D)** tests.

Considering the data relating to linear correlation, the highest value was found (r: 0.98; *p* < 0.0001) for the SRCW test, and the lowest value (r: 0.91; *p* < 0.0002) for the test RSP. Considering the intraclass correlation coefficient, the highest value was found. Figure 2 presents the reproducibility between measures of functional autonomy tests.

Observing the data presented, the reproducibility and agreement between the functional autonomy tests varied between the lowest value of 0.07 ± 0.74 BIAS (−1.38–1.54) for the RVDP test (Figure 2C) and the highest value of 0.32 ± 1.89 BIAS (−3.38–4.04) for the SRCW test (Figure 2D).

## Discussion

The objective of the present study was to analyze the reproducibility and agreement in applying four functional autonomy tests face-to-face and virtually for older women. This study found favorable agreement between the information collected in both assessments. There was a high rate of agreement between the application of the tests in the face-to-face and virtual conditions applied by the same evaluator, which makes this diagnostic assessment a reliable alternative for evaluating the functional autonomy of the older person in a virtual environment.

Functional autonomy is a health variable that is primarily strengthened throughout life by moving daily, which is considered a form of physical activity. By carrying out the tasks of daily life, the body shifts from a resting state and generates caloric expenditure (22).

With the advent of the SARS-CoV-2 pandemic, the decrease in these activities was linked to health problems and neurological, physiological, and functional declines inherent to aging and confinement, bringing about a need for virtual connections and online data collection (28).

Precautionary measures and social isolation imposed limitations on face-to-face activities, making it challenging to collect data through field trips, resulting in the need to think about alternative methods to the face-to-face ones, thus reaching the virtual environment (29), recommended by American College Sports Medicine [ACSM, 2002] (30), which sought to help and provide tools for people to practice their physical activities at home, using virtual technologies, which are not new, but still promising and with a wide range of applications (31, 32).

Due to its nature as a sustainable and easily accessible test, the virtual intervention model is widespread among older people, especially those with progressive diseases, presenting good results in effectiveness, adherence, easy applicability, feasibility, and autonomy (33–35).

Many studies report on the practice of physical exercise programs at home; however, the literature is scarce regarding the use of diagnostic assessments to support the prescription and supervision of training programs. These assessments are essential for ensuring the success of the results, which, according to Lacroix et al. (34) are superior when the programs are supervised, particularly in outcomes related to balance, strength, and muscular power.

Kis et al. (35), in a systematic review with meta-analysis, observed that supervised home training increased the adoption of exercise programs. The justification for these results is that even under minimal supervision, older people perform exercises with better quality, more attention, at a greater volume, and with more intensity. These characteristics improve cognition, improving the executive function of the physical movements (34).

Corroborating the statements mentioned above, the physical function of older people must be assessed using quantitative measures (36–38), which can be carried out in different settings, such as health promotion centers, clinics, public parks, or at home through digital health intervention, conceptualized by World Health Organization (39) as the use of digital, mobile, and wireless technologies to support the achievement of health objectives.

Since diagnostic assessment is essential for identifying the parameters of the variables involved in functional fitness, for the correct and accurate prescription of the training program (6), and following satisfactory reproducibility and agreement indices, as presented in the results of this study, the application of the evaluation of the functional autonomy tests W10M, RSP, SRCW, and RVDP, enables and reassures the application of these in the older adult population, in a precise, fast, safe and low-cost way. It is also worth noting that performing exercises in the home environment can favor older people’s adherence since, in this context, overcoming barriers such as fear of falling and fear of privacy is minimized (40).

When observing the consistency of values between the first and second evaluation of the functional autonomy tests used, the rising from sitting position (RSP) test, which aims to evaluate the functional capacity of the lower limbs (41), showed greater accuracy in face-to-face and virtual assessments, which can be attributed to activities carried out in daily life resulting from the movement of squatting and walking (42–45).

The data relating to the linear correlation and the intraclass correlation coefficient from the SRCW test, which assesses agility, dynamic, and recovered balance (26), showed a higher correlation between the others. Sitting and standing are considered one of the most important measures of physical capacity and one of the most demanding functional tasks from a biomechanical point of view (46, 47). However, the tests carried out by the older adults group are similar to activities of daily living, facilitating the assimilation of the evaluators’ verbal commands in the virtual scenario, thus allowing the use of the proposed evaluation carried out remotely, online, and supervised.

The analysis by Bland and Altmann (48) showed agreement between the assessments carried out face to face and virtually since most of the intersections between the bias and the average values were within the limits of agreement. The reproducibility and reliability analysis presented coefficients of variation with values varying between the lowest value of 0.07 ± 0.74 BIAS (−1.38–1.54) for the RVDP test and the highest value of 0.32 ± 1, 89 BIAS (−3.38–4.04) for the SRCW test. According to Morrow et al. (49), the lower the coefficient of variation, the greater the reproducibility of the protocol. Considering this aspect, it is believed that the variability of the time to complete the SRCW test, which evaluates the participant’s agility and balance can be attributed to the characteristics of the test itself, as it is the longest to perform among all the tests. The other tests, being shorter, allow for greater variability in the results (50), additionally, the participants presenting different levels of physical fitness may have been another contributing factor to this variation.

Alves et al. (51) mentioned that physical fitness and functional capacity are interconnected with advancing age, as they reflect the ability of older people to carry out activities of daily living within their environment, with autonomy. Given that the SRCW test involves movements related to these skills, the observed variability in test execution time among study participants can be justified.

We believe that the results of the present study provide good perspectives for physical and functional assessments of older people. The reproducibility of virtually performed tests facilitates the work of private clinics and public health workers, increasing the possibility of measuring functional health data for older people at a low cost.

The present study presents as a relevant point the originality of applying the functional autonomy test in virtual mode and its reproducibility and reliability. As the study participants were female, with no male participants, and only one evaluator applied the functional autonomy test to older women, we consider this fact a limitation of the study. Therefore, it is recommended that new evaluations be carried out using older men and that more than one evaluator be applied to the test.

## Conclusion

According to the results presented, there were no significant changes between V and FF and no significant changes between tests considering FF and V conditions. Additionally, we identified a significant intraclass correlation coefficient for the SRCW and RSP. The tests used in the present study showed good reproducibility and agreement in older people when carried out face to face and virtually. However, more studies are needed to investigate the reproducibility and effectiveness of the applicability of diagnostic assessments of the multiple variables that involve training programs. Furthermore, it is recommended that this study be replicated with different evaluators, male participants, and age groups.

## Data Availability

The raw data supporting the conclusions of this article will be made available by the authors under reasonable request.
